# Broad and potent antiviral activity of the NAE inhibitor MLN4924

**DOI:** 10.1038/srep19977

**Published:** 2016-02-01

**Authors:** Vu Thuy Khanh Le-Trilling, Dominik A. Megger, Benjamin Katschinski, Christine D. Landsberg, Meike U. Rückborn, Sha Tao, Adalbert Krawczyk, Wibke Bayer, Ingo Drexler, Matthias Tenbusch, Barbara Sitek, Mirko Trilling

**Affiliations:** 1 Institute for Virology, University Hospital Essen, University Duisburg-Essen, Essen, Germany; 2 Medizinisches Proteom-Center, Ruhr Universität Bochum, Bochum, Germany; 3 Institute for Virology, University Hospital Düsseldorf, Heinrich-Heine-University, Düsseldorf, Germany; 4 Department of Molecular and Medical Virology, Ruhr-University Bochum, Bochum, Germany

## Abstract

In terms of infected human individuals, herpesviruses range among the most successful virus families. Subclinical herpesviral infections in healthy individuals contrast with life-threatening syndromes under immunocompromising and immunoimmature conditions. Based on our finding that cytomegaloviruses interact with Cullin Roc ubiquitin ligases (CRLs) in the context of interferon antagonism, we systematically assessed viral dependency on CRLs by utilizing the drug MLN4924. CRL activity is regulated through the conjugation of Cullins with the ubiquitin-like molecule Nedd8. By inhibiting the Nedd8-activating Enzyme (NAE), MLN4924 interferes with Nedd8 conjugation and CRL activity. MLN4924 exhibited pronounced antiviral activity against mouse and human cytomegalovirus, herpes simplex virus (HSV)- 1 (including multi-drug resistant clinical isolates), HSV-2, adeno and influenza viruses. Human cytomegalovirus genome amplification was blocked at nanomolar MLN4924 concentrations. Global proteome analyses revealed that MLN4924 blocks cytomegaloviral replication despite increased IE1 amounts. Expression of dominant negative Cullins assigned this IE regulation to defined Cullin molecules and phenocopied the antiviral effect of MLN4924.

Several viruses exploit the ubiquitin (Ub) and/or the proteasome system to regulate the stability of viral proteins or to induce proteolysis of cellular restriction factors^1^,^2^. Consistently, interference with the Ub conjugation system (e.g., by the drug PYR-41) or proteasome activity (e.g., by MG-132 or Bortezomib) diminishes the replication of several viruses (e.g.^3^,^4^,^5^,^6^). However, sequence-based searches revealed the existence of >600 genes coding for putative Ub ligases in the human genome^7^. Global Ub-conjugation profiling identified >650 Ub-conjugated proteins in HeLa cells^8^ and proteome wide assessments show that the half-life of >80% of proteins significantly increases upon inhibition of the proteasome^9^. Based on these findings, it is obvious that the blockade of ubiquitination and/or proteasome activity severely affects numerous cellular processes and provokes serious side effects. The fact that several viruses require the Ub proteasome pathway for their replication in conjunction with the toxicity of drugs targeting the global function of Ub conjugation and proteasomal degradation raises the question whether defined classes of Ub ligases can be targeted to treat viral infections. Thus, the identification of essential host factors implicated in discrete aspects of the Ub proteasome pathway constitutes a promising strategy for the discovery of novel antiviral drugs.

In this respect, a particular interesting family of Ub ligases are the Cullin Roc Ub ligases (CRL) which are composed of a Cullin backbone and the Ub ligase RocA. Humans encode seven different Cullin proteins (Cullin 1, 2, 3, 4A, 4B, 5 and 7)^10^. The activity of CRLs is controlled by the modification of the Cullin with the small protein Nedd8^11^. CRLs with a covalently linked Nedd8 are enzymatically active, whereas complexes lacking Nedd8 moieties are inactive. The first step of Nedd8 conjugation is catalysed by the enzyme *Nedd8 activating enzyme* (NAE). Quantification of Nedd8-conjugated proteins by mass-spectrometry revealed that besides Cullins some other proteins can also be neddylated. However, normalized spectral abundance factor (NSAF) determinations indicate that the contribution of Cullins to Nedd8-conjugated proteins approaches 100%^11^. Recently, a highly specific first-in-class NAE inhibitor, namely MLN4924, has been described^12^. MLN4924 activity and toxicity have been thoroughly studied *in vitro* and *in vivo* and MLN4924 is currently in clinical trials in patients (see http://clinicaltrials.gov/ct2/results?term=MLN4924&Search=Search) as a treatment for malignancies. A phase I trial observed that common side effects were fatigue and nausea and ≥15% of patients reported adverse events but grade 4 adverse events and treatment-related deaths did not occur^13^.

We have previously shown that the mouse cytomegalovirus (MCMV)-encoded protein pM27 exploits *DNA-damage and DNA-binding* (DDB) 1 containing Cullin 4A/B ubiquitin ligase complexes to induces (poly-) ubiquitination and subsequent proteasomal degradation of STAT2^14^.

Here, we show that MCMV replication is severely compromised in the presence of MLN4924, displaying viral dependency on NAE and CRLs. Besides the effect on MCMV, we found potent antiviral activity of MLN4924 against the human-pathogenic viruses HCMV, Herpes simplex virus (HSV)-1, HSV-2, adenovirus 5 (AdV5) and influenza virus PR8, but not against vaccinia virus (VACV) and vesicular stomatitis virus (VSV). Our findings uncover NAE and CRLs as essential and druggable host factors for the replication of several clinically relevant viruses.

## Results

### Pharmacological blockade of NAE acts antiviral against cytomegaloviruses

**Figure 1:**
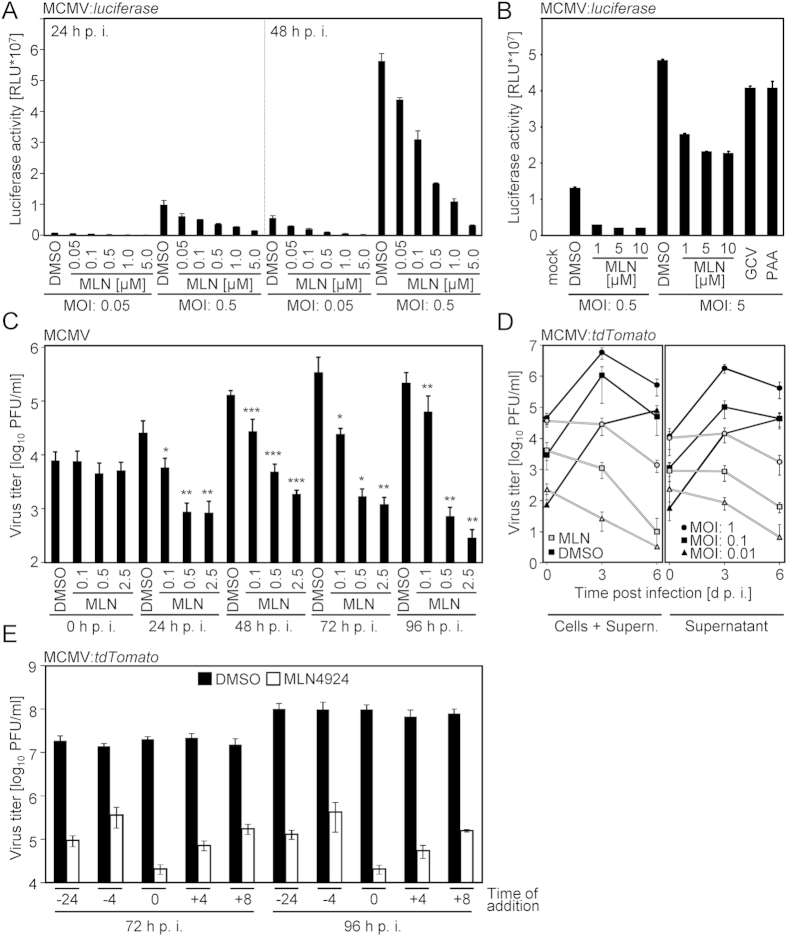
MLN4924 exerts antiviral activity against MCMV. (**A**) NIH3T3 cells were treated with indicated concentrations of MLN4924 (0.05–5 μM) and simultaneously infected with Δm157-MCMV:*luciferase* (multiplicity of infection [MOI] 0.05 or 0.5). At 24 (left panel) or 48 (right panel) h post infection (h p. i.) cells were lysed and luciferase activity was determined. Relative light units (RLU) are depicted. (**B**) Cells were treated with indicated concentrations of MLN4924 (1–10 μM) or DMSO and infected with Δm157-MCMV:*luciferase* (0.5 [left panel] or 5 PFU/cell [right panel]). MLN4924 treatment was compared to treatment with 50 μM Ganciclovir (‘GCV’) and 250 μg/ml phosphonoacetic acid (‘PAA’), respectively. Luciferase activity was determined 1 d p. i. (**C**) NIH3T3 cells were treated with DMSO or MLN4924 (0.1–2.5 μM) and infected with MCMV (0.05 PFU/cell). At 0, 24, 48, 72 and 96 h post infection, samples were frozen. MCMV titers were determined by plaque titration. The experiment was performed in triplicates which were each titrated in duplicates (n = 3 * 2). Shown is the arithmetic mean (AM) with the standard deviation (SD). (**D**) Immortalized C57BL/6 fibroblasts were treated with 2.5 μM MLN4924 and infected with 0.01, 0.1 or 1 PFU/cell Δm157-MCMV:*tdTomato*. At 0, 3 and 6 d p. i., cells and supernatants were frozen and titers were determined by the titration of red fluorescent foci. The experiment was conducted in duplicates and each well was titrated in triplicates (n = 2 * 3). AM with SD is depicted. (**E**) Mouse fibroblast cells were treated with DMSO or 2.5 μM MLN4924 starting 24 or 4 h prior to infection, at the time of infection and at 4 or 8 h post infection, respectively. Δm157-MCMV:*tdTomato* titers were determined at 3 and 4 d p. i. For each setting, at least 3 plaque titrations were performed. AM with SD is depicted.

We raised the question whether CRL activity is needed for efficient replication of MCMV. To test this, NIH3T3 fibroblasts were treated with graded concentrations of MLN4924 and infected with a recombinant MCMV harbouring a *luciferase* reporter gene under the control of the endogenous MCMV *m157* promoter/enhancer. In this construct, reporter gene expression occurs with *early*/*late* kinetics allowing an evaluation of the antiviral effects which are manifested at this or earlier steps of the replication cycle. At 24 h and 48 h post infection, cells were lysed and reporter gene expression was quantified. A dose-dependent decrease of luciferase activity was observed upon MLN4924 treatment (Fig. 1A). For comparison, Ganciclovir (GCV) and phosphonoacetic acid (PAA; a pyrophosphate mimetic [like Forscarnet]), two well-known compounds affecting cytomegaloviral replication, were included in the experiment. Intriguingly, MLN4924 reduced luciferase expression more effectively than GCV and PAA (Fig. 1B). This finding prompted us to test whether MLN4924 elicits antiviral activity in terms of reducing MCMV progeny. MCMV-permissive cells were treated with different concentrations of MLN4924 and infected with wt-MCMV. At 1, 2, 3, and 4 days post infection, viral titers were determined by plaque titration. MLN4924 reduced MCMV titers significantly and dose-dependently (Fig. 1C), indicating that NAE activity is required for MCMV replication. This profound antiviral activity was observed irrespective of the initial virus inoculum (0.01, 0.1 and 1 PFU/cell) and in terms of a reduction of cell-associated as well as supernatant virus (Fig. 1D). Time-of-addition experiments revealed significant antiviral activity of MLN4924 even when the drug was administered at 4 or 8 hours after infection, indicating that a step beyond viral entry is affected (Fig. 1E).

**Figure 2:**
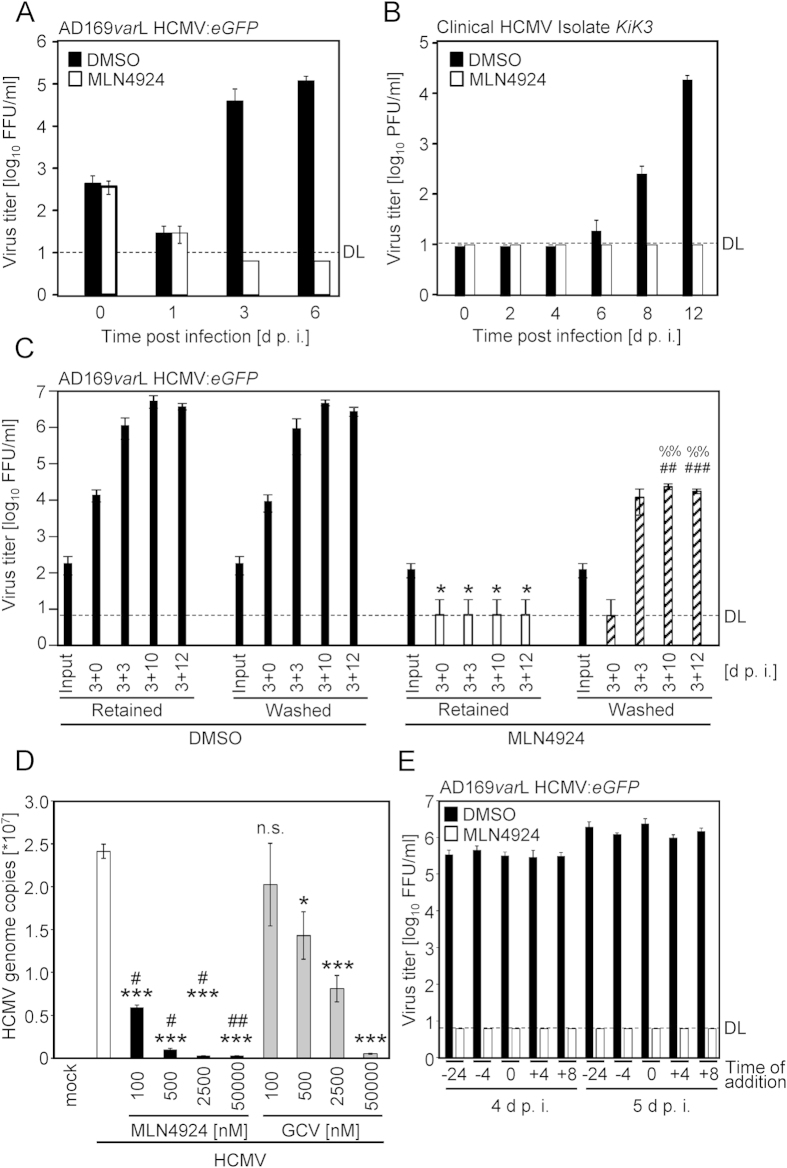
MLN4924 exerts antiviral activity against human CMV. (**A**) DMSO- or MLN4924-treated (1 μM) MRC-5 cells were infected with an eGFP-expressing AD169*varL* HCMV (0.1 PFU/cell). Cells were frozen at the indicated time points post infection. Viral titers were determined by titration of GFP-positive foci. Three independent samples were determined per setting (n = 3). AM with SD is depicted. (**B**) As in (A), but cells were treated with 2.5 μM MLN4924, infected with a clinical HCMV isolate, titers were determined by plaque titration and n = 2 * 3 replicates were assessed. (**C**) DMSO- or MLN4924-treated (2.5 μM) MRC-5 cells were infected with AD169*varL* HCMV:*eGFP* (0.1 PFU/cell). After 3 days, cells were either vigorously washed to withdraw MLN4924 or the medium (containing either DMSO or MLN4924) was retained throughout the experiment. At the indicated times post infection, virus titers were quantified. Three independent replicates were determined per setting (n = 3). AM with SD is depicted. (‘*’indicates statistical significance compared to retained DMSO-treated cells; ‘%’ indicates statistical significance compared to ongoing MLN4924 treatment and ‘#’ indicates statistical significance compared to washed DMSO-treated cells). (**D**) MRC-5 cells were treated with indicated concentrations of MLN4924 or ganciclovir (‘GCV’) and infected with HCMV. At 3 d post infection, viral genome copies were quantified. Asterisks indicate statistical difference compared to untreated cells and hash symbols indicate the statistical difference compared to corresponding GCV concentrations. (**E**) MRC-5 cells were treated with DMSO or 2.5 μM MLN4924 starting at 24 or 4 h prior to infection, at the time of infection and at 4 or 8 h post infection, respectively. HCMV titers were determined at 4 and 5 d p. i. For each setting, at least 3 plaque titrations were performed. AM with SD is depicted.

Next, antiviral activity of MLN4924 against human cytomegalovirus was assessed. HCMV-permissive MRC-5 cells were treated with 1 or 2.5 μM MLN4924 and infected with two different AD169-HCMVs (AD169*var*S and AD169*var*L, respectively^15^). Both concentrations suppressed replication of HCMV AD169*var*S (data not shown) and AD169*var*L (Fig. 2A) at 3 and 6 d p. i. below the detection limit, whereas DMSO-treated cells allowed efficient HCMV replication. MLN4924 was also effective against the newly established (only 3 *in vitro* passages) clinical HCMV isolate *KiK3* (Fig. 2B). To test the reversibility of MLN4924-dependent effects, continuous treatment was compared to treatment for the initial 3 days of replication followed by withdrawal of MLN4924. No HCMV replication could be detected in continuously MLN4924-treated cells (Fig. 2C). Withdrawal of MLN4924 after 3 days of replication resulted in HCMV titers that were significantly higher than those in cells under continuous MLN4924 treatment, but still significantly lower than the viral progeny in DMSO-treated cells (Fig. 2C). This indicates that MLN4924 has a potent antiviral activity against HCMV and that its effect is partially reversible. The reversible nature of the antiviral activity is incompatible with the interpretation that MLN4924 acts antiviral by inducing cell death.

Since viral genome copy numbers represent a clinically relevant parameter for the assessment of viral propagation, we determined viral genome amplification in the presence and absence of MLN4924, respectively. For comparison, treatment with the clinically approved drug GCV was simultaneously performed. MLN4924 significantly suppressed viral genome replication even at nanomolar concentrations and significantly outperformed GCV at each concentration (Fig. 2D). Our data uncover that NAE activity and/or its effect on CRL activity constitutes an essential host determinant for CMV genome amplification and replication. Consistent with the results obtained for MCMV and the notion of a post entry effect, pronounced antiviral activity was observed even when the drug was added at 4 or 8 hours after HCMV infection (Fig. 2E).

### MLN4924 acts antiviral against Herpes simplex virus 1 and 2

**Figure 3:**
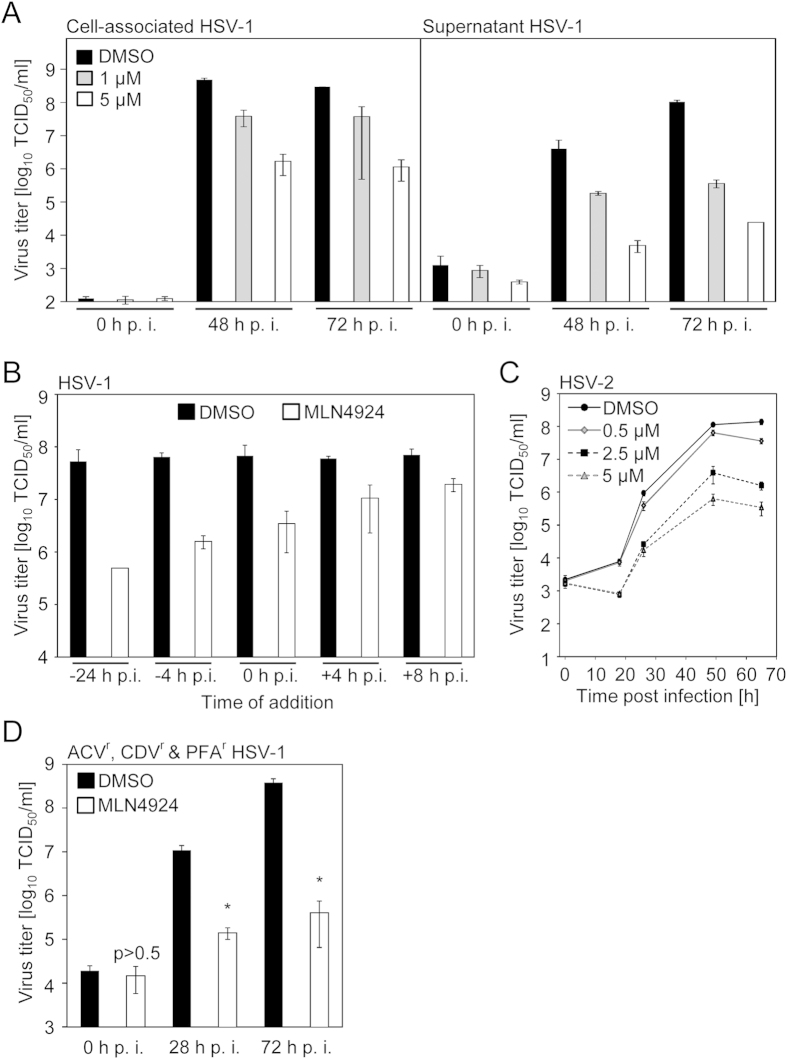
MLN4924 exerts antiviral activity against human Herpes simplex virus 1 and 2. (**A**) DMSO- (black bars) or MLN4924-treated (1 μM [grey bars] or 5 μM [open bars]) Vero cells were infected with HSV-1 strain F (5000 PFU/well). At 0, 48 and 72 h p. i. TCID50 titers (12 row format) of infected cells (left panel) and the supernatant (right panel) were determined. For each setting, 2 independent TCID50 determinations were performed. AM with SD is depicted. (**B**) Vero cells were treated with DMSO or MLN4924 (2.5 μM) starting at 24 or 4 h prior to infection, at the time of infection and at 4 or 8 h post infection, respectively. HSV-1 TCID50 titers were determined at 36 h p. i. For each setting, 3 TCID50 measurements were performed. AM with SD is depicted. (**C**) Vero cells were treated with DMSO or indicated concentrations of MLN4924 and infected with HSV-2. After the indicated times, virus titers were determined. For each setting 3 independent measurements were performed (n = 3). AM with SD is depicted. (**D**) DMSO- or MLN4924-treated Vero cells were infected (5000 PFU/well) with a clinical HSV-1 isolate resistant against acyclovir (ACV), cidofovir (CDV) and foscarnet (PFA) described in^17^. At 0, 28 and 72 h p. i. TCID50 titers were determined. For each setting, 3 independent TCID50 determinations were performed. AM with SD is depicted.

To test whether the requirement for NAE and CRL activity is restricted to members of the beta-herpesvirus family, antiviral potency against HSV-1 was assessed. HSV-1 replication was determined in Vero cells in the presence of MLN4924 or DMSO. Cell-associated and extracellular virus titers were quantified. MLN4924 reduced the cell-associated virus by more than 100-fold and the supernatant virus even by close to 4 orders of magnitude (Fig. 3A), indicating that HSV-1 is also highly susceptible to pharmacological interference with NAE and Cullin activity. This finding also suggested that MLN4924 does not require type I IFN, since Vero cells harbour deletions comprising type I IFN genes^16^.

A time-of-addition experiment revealed that pre-incubation with MLN4924 potentiated the antiviral effect against HSV-1 (Fig. 3B). When MLN4924 was administered at 4 and 8 hours respectively post HSV-1 infection, virus titers were reduced by more than 80% and 70%, respectively (Fig. 3B), strongly suggesting that MLN4924 elicits at least a part of its effect after attachment and entry. As expected from the high degree of homology between these two viruses, HSV-2 replication was also found to be MLN4924-sensitive (Fig. 3C).

### MLN4924 is effective against multidrug resistant HSV-1 isolates

Due to the fact that the mode of action of MLN4924 differs from the mechanisms of all anti-herpesviral drugs currently used in clinics, we hypothesized that even multidrug-resistant clinical HSV-1 isolates should be susceptible to MLN4924. To this end, the potency of MLN4924 against a multidrug (Aciclovir-, Cidofovir- and Foscarnet-) resistant clinical HSV-1 isolate (described in^17^) was tested. Indeed, MLN4924 significantly reduced the viral replication (Fig. 3D). Taken together, our findings uncover NAE and CRLs as novel host factors for clinically relevant viruses (e.g. HCMV and HSV-1) amenable to pharmacological intervention even upon established multidrug resistance.

### Adenovirus and influenza virus are MLN4924 susceptible whereas vesicular stomatitis virus and vaccinia virus are not

**Figure 4:**
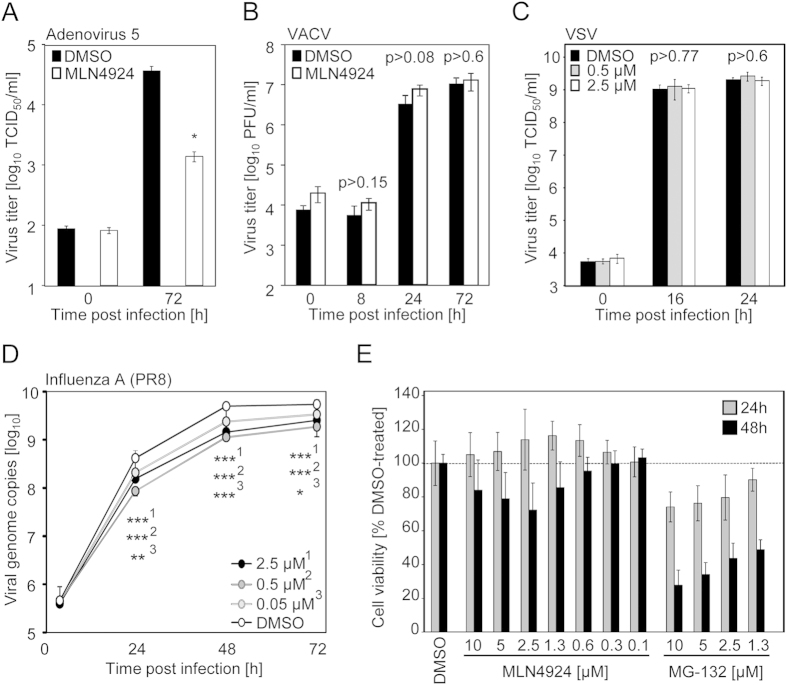
MLN4924 exerts antiviral activity against Adenovirus 5 and Influenza virus PR8, but not against VSV or Vaccinia virus. (**A**) DMSO- or MLN4924-treated (2.5 μM) Vero cells were infected with Adenovirus 5 (1000 PFU/well). At 0 and 72 h p. i. TCID50 titers were determined. For each setting, 3 independent TCID50 replicates were performed. AM with SD is depicted. Similar results were obtained on A549 cells (data not shown). (**B**) CV-1 cells were treated with DMSO or MLN4924 (2.5 μM) and infected with VACV strain WR at MOI 0.01. Virus titers (PFU/ml) were determined at indicated time points after infection. For each setting, 3 measurements were conducted. AM with SD is depicted. (**C**) Vero cells were treated with DMSO or MLN4924 and infected with VSV (500 PFU/well). After the indicated times (0, 16 and 24 h p. i.), TCID50 titers were determined. For each setting, 4 independent measurements were performed (n = 4). AM with SD is depicted. (**D**) MDCK cells were treated with indicated MLN4924 concentrations and infected with Influenza virus PR8 (2000 PFU/well). At the indicated time points (4, 24, 48 and 72 h p. i.) supernatants were harvested and viral genome copies were determined by qPCR. For each setting, 3 measurements were conducted. AM with SD is depicted. (**E**) Vero cells were treated for 24 or 48 h with DMSO or indicated concentrations of MLN4924 and MG132, respectively. Cell viability was assessed using Rotitest Vital according to manufacturer’s instructions. At least 9 independent measurements were performed. AM with SD is depicted.

To test whether the antiviral activity of MLN4924 is restricted to the genus herpesviruses, the activity against AdV5 was studied. Treatment of A549 cells (data not shown) and Vero cells (Fig. 4A) with MLN4924 significantly reduced AdV5 titers. The phylogenetically even more distant viruses, vesicular stomatitis virus (VSV; a member of the *Rhabdoviridae*), influenza virus PR8 (a member of the *Orthomyxoviridae*) and vaccinia virus (VACV, a member of the *Poxviridae*) were also evaluated. VACV replication in CV-1 cells was not reduced by MLN4924 treatment (Fig. 4B), indicating a degree of specificity. The cytopathic appearance upon proceeding VSV replication was virtually indistinguishable between MLN4924- and DMSO-treated control cells (data not shown). Consistently, VSV titers remained unaffected by MLN4924 (Fig. 4C). Conversely, the replication of Influenza PR8, as determined by the increase in viral genome copies, was significantly affected by MLN4924 (Fig. 4D), but the magnitude of inhibition was less pronounced as compared to the tested herpesviruses. Altogether, these findings suggest that certain virus families require neddylation-dependent CRL activity whereas others do not. MLN4924 elicits strong antiviral activity against certain RNA and several herpesviruses.

### MLN4924 exhibits less cytotoxicity than inhibitors of the proteasome

The inability of MLN4924 to elicit antiviral activity against some viruses (VSV and VACV - see Fig. 4) and the partial reversibility of the antiviral effect against HCMV (see Fig. 2C) already suggested that MLN4924 does not simply act by causing cell death. In contrast to the global inhibition of all proteasomal degradation events (e.g. by bortezomib and MG-132), the activity spectrum of MLN4924 is restricted to CRLs and it is well-tolerated *in vivo*^18^. Nevertheless, we compared the effect of MLN4924 and MG-132 on cell viability after 24 and 48 h treatment. MLN4924 did not reduce cell viability during a 24 h treatment period and only slightly after 48 h of treatment. Consistent with the more restricted activity spectrum, MLN4924 had clearly less detrimental effects on cell viability than the broad spectrum proteasome inhibitor MG-132 (Fig. 4E).

### Pharmacological blockade of CRL activity reverts STAT2-degradation in MCMV-infected cells

The MCMV-encoded IFN antagonist pM27 exploits DDB1-containing CRLs to instruct (poly-) ubiquitination and subsequent proteasomal degradation of the transcription factor STAT2^14^. Consistently, STAT2 degradation depends on viral gene expression upon MCMV infection - as shown by the abrogation upon UV-irradiation of the inoculum virus - and the presence of the gene *M27* (Supplementary Fig. 1A). Treatment with MLN4924 increased STAT2 amounts in wt-MCMV-infected cells as compared to DMSO-treated or untreated control cells (Supplementary Fig. 1A). To exclude that STAT2 recovery is due to alterations of pM27 amounts, an experiment was performed using M27HA-MCMV and graded MLN4924 concentrations. MLN4924 treatment restored STAT2 without lowering pM27-HA levels (Supplementary Fig. 1B). Taken together, these data indicate that a blockade of CRL activity by the inhibition of NAE, the first enzyme of the Nedd8 activation and conjugation pathway, counteracts STAT2 degradation mediated by the MCMV-encoded IFN antagonist pM27.

### The antiviral activity of MLN4924 did not rely on IFN induction

A blockade of viral interferon antagonism could have explained the antiviral activity against cytomegaloviruses. To study potential effects of MLN4924 on IFN signalling, an NIH3T3-based IFN-reporter cell line harbouring a luciferase reporter gene under the control of an IFN-stimulated response element (ISRE) was treated with recombinant IFN-α or IFN-γ in the presence or absence of MLN4924. Inhibition of NAE and CRLs did not markedly change IFN signal transduction (Supplementary Fig. 1C). Another potential explanation for the antiviral effect of MLN4924 could be increased IFN synthesis and the establishment of an antiviral state executed by IFN-stimulated gene products. To test this, supernatants of MLN4924-conditioned cells were applied to the IFN reporter cell line. For comparison, grading concentrations of recombinant mouse IFN-α were administered to the reporter cells. Two units (U) rec. IFN-α per ml sufficed to significantly activate the ISRE promoter/enhancer (Supplementary Fig. 1D - left panel). No increase of ISRE-driven luciferase activity was elicited by conditioned media of MLN4924-treated cells (Supplementary Fig. 1D - right panel) excluding that MLN4924 induces IFN concentrations above 2 U/ml. Previous work conducted by us and others showed that IFN concentrations below 2 U/ml fail to suppress CMV replication to the extent observed upon MLN4924 treatment (19 and data not shown). Thus, the antiviral activity of MLN4924 could hardly be explained by IFN induction. MLN4924 counteracts the MCMV-encoded IFN antagonist pM27 (see Supplementary Fig. 1A). In cell culture, ΔM27-MCMV replication proceeds wt-like in the absence of exogenously added IFN^14^,^20^,^21^. Therefore, the importance of CRL activity must go beyond pM27-mediated STAT2 degradation. Consistently, ΔM27-MCMV was also susceptible to MLN4924 (Supplementary Fig. 1E).

To formally exclude that the type I IFN system mediates the activity of MLN4924, its antiviral potency against MCMV was evaluated in immortalized IFNAR1-deficient fibroblasts. MLN4924 was fully proficient to suppress MCMV replication in the absence of IFNAR1 (Supplementary Fig. 1F). The experiment was repeated on primary murine embryonic fibroblasts (MEF) from C57BL/6 and IFNAR1^−/−^ mice, but no significant difference between the cell types was observed at 2, 4 and 6 days post infection (p > 0.66, p > 0.79 and p > 0.23, respectively - data not shown). These findings indicate that although MLN4924 counteracts the activity of the interferon antagonist pM27, it elicits its antiviral activity against MCMV pM27- and type I IFN-independently, suggesting that NAE and CRL activity are required for further layers of protein turnover regulation during viral replication.

### Global proteome analysis revealed that MLN4924 blocks MCMV replication despite increased levels of IE proteins

**Figure 5:**
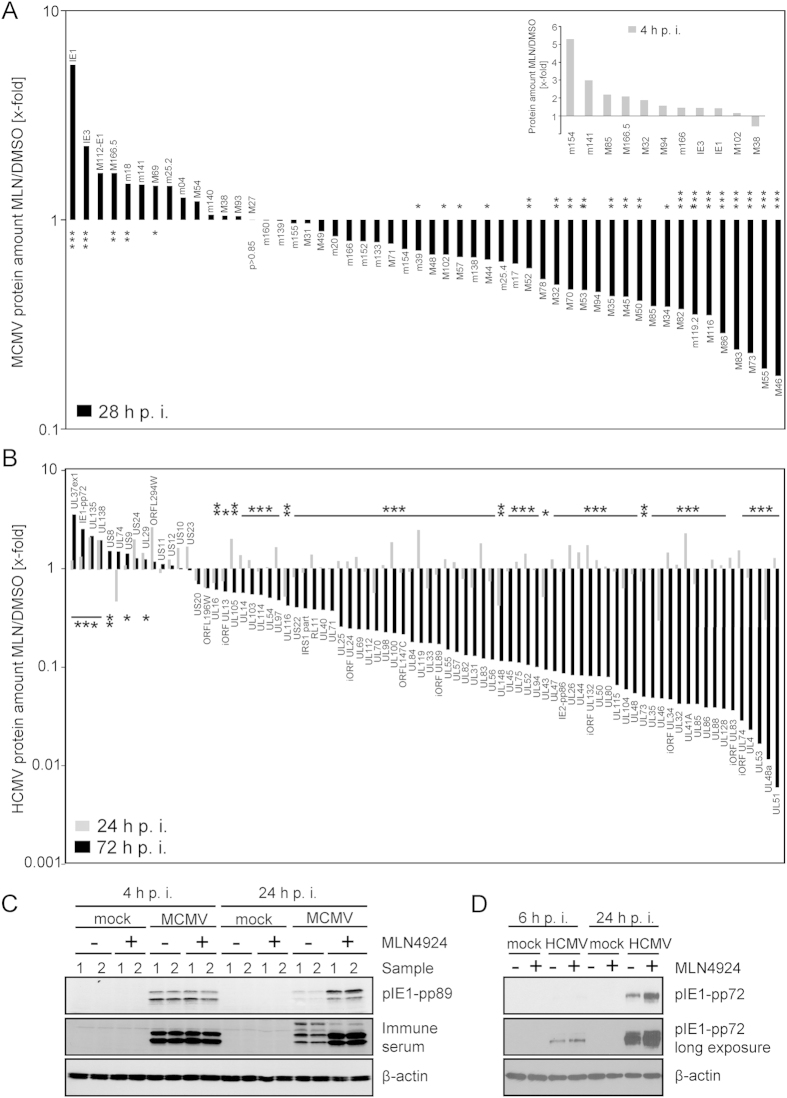
Proteome profiling of cytomegalovirus-infected cells reveals antiviral activity despite increasing IE protein amounts. (**A**) DMSO- or MLN4924-treated (2.5 μM) NIH3T3 cells were infected with wt-MCMV (10 PFU/cell). At 4 and 28 h p. i., cells were lysed and subjected to mass-spectrometry-based label-free quantitative proteome analysis. The following numbers indicate the independent replicates for each setting: mock DMSO (n = 5), mock MLN4924 (n = 6), MCMV 4 h p. i. DMSO (n = 6), MCMV 4 h p.i. MLN4924 (n = 4), MCMV 28 h p.i. DMSO (n = 6) and MCMV 28 h p.i. MLN4924 (n = 6). Relative changes in the abundance of indicated MCMV-encoded proteins are depicted. (**B**) DMSO- or MLN4924-treated (2.5 μM) MRC-5 cells were infected with HCMV AD169*var*L (3 PFU/cell). At 6, 24 and 72 h p. i., cells were lysed and subjected to mass-spectrometry-based quantitative proteome analysis. For MLN4924-conditioned mock cells, n = 3 independent samples were analysed. For each of the other 11 settings, n = 6 independent replicates were performed. Relative changes in the abundance of indicated HCMV-encoded proteins after 6h (see Suppl. Table 2), 24 h (grey bars) or 72 h (black bars) are indicated. (**C**) As in (**A**), but the lysates were subjected to immunoblot analysis using the indicated antibodies or MCMV immune serum. (**D**) Cells were treated with MLN4924 and infected for 6 or 24 h with HCMV. Cell lysates were normalized and subjected to immunoblotting using indicated antibodies.

To get insights into the molecular mechanism behind the antiviral activity of MLN4924 against MCMV, a global analysis of the proteome was performed by label-free quantification using liquid chromatography and mass spectrometry (LC-MS). Cells were treated with MLN4924 and infected with MCMV. At 4 and 28 h post infection, cells were lysed and subjected to MS analysis. In total, 33 independent global proteome profiles were determined and 4115 proteins were quantified. Comparing MLN4924- and DMSO-treated cells upon MCMV infection, the abundance of 842 host proteins changed significantly (ANOVA p < 0.05) - 319 of them more than 2-fold (Suppl. Table 1). Of the 842 proteins, the expression of 399 increased (Suppl. Table 1). Consistent with our immunoblot analysis (Supplementary Fig. 1), the unbiased global proteome profiling data set confirmed a significant restoration of STAT2 (but not STAT1 and STAT3) in MCMV-infected cells upon MLN4924 treatment (Suppl. Table 1). In addition to the host proteins, 52 viral proteins were quantified. At 4 h post infection, MLN4924 treatment resulted in a significantly increased abundance of 10 MCMV-encoded gene products (m154, m141, M85, m166.5, M32, M94, m166, IE3, IE1 and M102 - in descending fold regulation [5.28- to 1.15-fold]) and the abundance of only one MCMV protein was slightly decreased (M38; 0.42-fold expression) (Fig. 5A inset). This excludes negative effects of MLN4924 on viral attachment, entry, unpackaging, transport of the viral capsid to the nucleus, insertion of the DNA and initiation of *immediate early* (*IE*) gene expression. At 28 h post infection, the abundance of the IE proteins pIE1-pp89 and pIE3 was even more increased, whereas numerous other MCMV proteins were significantly down-regulated (Fig. 5A). Consistent with the M27-HA immunoblot data (Supplementary Fig. 1B), pM27 levels remained unaffected by MLN4924 treatment (Fig. 5A). The strongest suppressive effect was observed for the minor capsid protein pM46 and for pM55-gB (Fig. 5A). Thus, the proteome profiling data are compatible with the decreased abundance of essential viral proteins in MLN4924-treated cells and overall suppressed viral replication at a step beyond immediate early protein expression.

### MCMV infection does not interfere with the Cullin ubiquitin ligase composition within host cells

The γ-herpesvirus Epstein-Barr virus (EBV, HHV-4, TaxID 10376) interferes with CRL activity via BPLF1-dependent degradation of nuclear Cullin proteins^22^. A similar effect in cells infected with the β-herpesvirus MCMV would be hardly compatible with the interpretation that CRLs constitute relevant host factors for cytomegaloviruses. Therefore, we examined the proteome data set concerning a potential deregulation of proteins directly or indirectly related to CRL activity. None of the proteins involved in neddylation and the CRL pathway was significantly down-regulated, some were even slightly up-regulated in MCMV-infected cells (Supplementary Fig. 2A).

To directly address CRL activity in MCMV-infected cells, the protein amount of p21 (CIP1/WAF1) was assessed. p21 is a well-known target of Cullin-1/Skp1/Skp2^23^ and Cullin-4/Cdt2 ubiquitin ligase^24^. While preserved CRL activity keeps p21 amounts continuously low, a general interference with CRL activity in MCMV-infected cells would stabilize p21 due to lacking proteasomal degradation. In contrast to EBV-positive cells^22^, p21 did not increase upon MCMV infection (see untreated or DMSO-treated cells - Supplementary Fig. 2B). Pharmacological interference with the CRL activity by MLN4924 led to the stabilization of p21 in mock- and MCMV-infected cells (Supplementary Fig. 2B). Thus, p21 levels serve indeed as surrogate marker for global CRL activity. This finding rules out a global impairment of CRL functions in MCMV-infected cells and highlights fundamental differences in the strategies of CMV and EBV in terms of regulating CRL activity.

### Global MS analysis revealed the footprint of inhibited CRL activity on the HCMV proteome

To examine the effect of MLN4924 on gene products expressed during HCMV replication, extensive global proteome analysis was conducted. Cells were either treated with MLN4924 or DMSO and infected with HCMV or left uninfected. Due to the slow replication of HCMV, three time points (6, 24 and 72 h post infection) were chosen for the analysis. For MLN4924-conditioned mock cells, n = 3 independent samples were analysed. For each of the other 11 settings, n = 6 independent replicates were performed. More than 4400 proteins were identified and more than 3250 different proteins including 81 viral proteins were quantified. At 6 h post infection, only a single HCMV protein (pUS24) was significantly (ANOVA p < 0.05) and more than two-fold reduced (Suppl. Table 2). Even at 24 h post infection, only 3 proteins were significantly and more than two-fold down-regulated (Fig. 5B and Suppl. Table 2) indicating that the effects on attachment, entry, capsid transport, unpackaging, DNA insertion into the nucleus and IE transcription are not responsible for the antiviral activity of MLN4924. After 72 hours, MLN4924 treatment significantly changed 70 HCMV-encoded proteins. Consistent with the antiviral activity of MLN4924, most viral proteins were significantly down-regulated - some more than 100-fold (Fig. 5B). Only 3 HCMV proteins showed significant and more than two-fold increase: pUL37ex1, pUL135 and pIE1-pp72. The increase in MCMV IE1-pp89 and HCMV pIE1-pp72 levels observed in the MS analysis was confirmed by immunoblotting (Fig. 5C,D).

Within the host proteins significantly increased by MLN4924 in HCMV-infected cells, STAT2 was found among the 40 most strongly regulated proteins. Although MLN4924 decreased STAT2 levels in mock-infected cells, a >4-fold increase (p < 0.0006) became evident in HCMV-infected cells (Supplementary Fig. 3A). To confirm this finding, an experiment was performed where MLN4924 was administered for shorter periods of time (2, 4, 8 and 24 h, respectively) to cells which had been previously infected for 64 h in the absence of the drug to minimize the effects of MLN4924 on viral protein expression. Even under such conditions, MLN4924 induced a partial recovery of STAT2 (Supplementary Fig. 3B). This suggests that the HCMV-encoded STAT2 antagonism is MLN4924-sensitive - as in the case of MCMV.

### Dominant negative Cullin mutants phenocopy the effect of MLN4924

**Figure 6:**
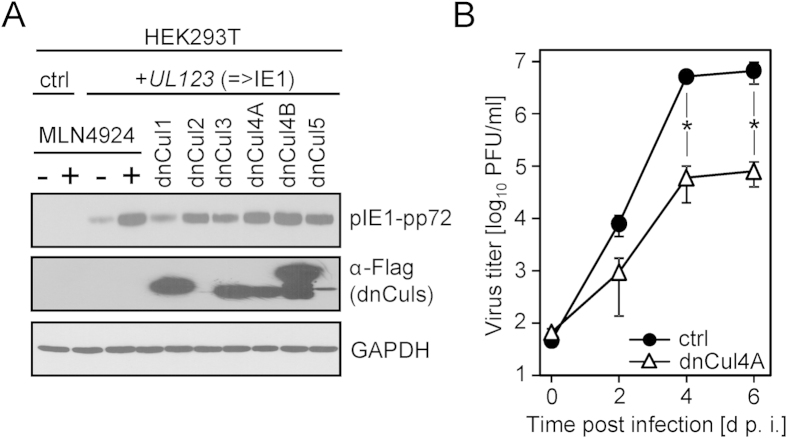
Dominant negative Cullins phenocopy the effect of MLN4924. (**A**) Cells were transfected with indicated dominant negative Cullins (dnCuls) for 24 h. Subsequently, cells were co-transfected with *UL123* expression vectors and the indicated dnCul. Cell lysates were normalized and subjected to immunoblotting. (**B**) Replication analysis of a recombinant MCMV expressing dnCul4A and a control (‘ctrl’) MCMV which expressed the fluorescent protein dsRed-Mito (Clontech).

Certain Cullins have long protein half-life times (~68 h for Cullin 5^25^) and others (e.g. Cullin 1 and 3) are essential for proliferation^26^,^27^,^28^. This limits the suitability of siRNA- and CRISPR/Cas9-based approaches. To exclude off-target effects of MLN4924, we used dominant negative Cullins (DnCul) which are immediately active upon expression and have been thoroughly studied on a global level by extensive proteome profiling^29^,^30^. The HCMV pIE1-pp72 stabilizing effect of MLN4924 was recapitulated by co-transfection of *UL123*-coding plasmids with dominant-negative Cullin truncation mutants (‘dnCul’). The most pronounced effect was elicited by inhibition of Cullin 4A/B, 2 and 5 (Fig. 6A). To analyze whether the antiviral activity of pharmacological inhibition of NAE und CRL activity could be similarly recapitulated using dnCuls, a recombinant MCMV mutant expressing dnCul4A was generated and its replication was compared with that of an MCMV expressing an irrelevant protein (here the fluorescent protein ‘dsRed-Mito’). Interference with Cul4A activity sufficed to significantly reduce MCMV replication more than 80-fold (Fig. 6B). Taken together, our data uncover CRLs as regulators of CMV IE Protein stability and as druggable host determinants not only essential for HCMV, but also for other clinically relevant viruses like HSV-1 and HSV-2.

## Discussion

MCMV and HCMV counteract IFN-induced signalling by targeting STAT2^14^,^21^,^31^,^32^. While MCMV accomplishes this task by pM27, the identity of the analogous HCMV protein is still elusive. By probing into the mechanism of pM27, the CRL4A/B adapter protein DDB1 was identified as a cellular interaction partner. To analyse whether CRL activity is required for STAT2 degradation, we used MLN4924, which inhibits CRL activity by forming a stable adduct with Nedd8 in the active centre of NAE^33^. Since Nedd8-conjugation to Cullins constitutes a switch for CRL activity, MLN4924 interferes with the function of CRLs. Consistent with the notion that enzymatic activity of CRLs is required for MCMV-induced STAT2 degradation, immunoblot experiments and an unbiased MS approach revealed the restoration of STAT2 amounts upon MLN4924 treatment in MCMV-infected cells (see Supplementary Fig. 1). MLN4924 also restored STAT2 levels in HCMV-infected cells, indicating that CRLs are also required for HCMV-induced STAT2 degradation. However, without knowledge concerning the identity of the HCMV-encoded antagonist, effects on the abundance of the antagonist itself and inhibitory effects on cellular co-factors cannot be unambiguously differentiated.

During these experiments, a striking effect of MLN4924 on MCMV became apparent: Viral replication was significantly and dose-dependently affected by MLN4924. MLN4924 also exhibited potent antiviral activity against HCMV, including low-passage clinical isolates. Based on the well-described molecular mechanism of MLN4924, this finding suggested that CMVs require NAE and CRL activity for their replication. Consistently, dominant negative Cullin truncation molecules recapitulated the MLN4924-sensitive regulation of pIE1-pp72 stability in transfection experiments and an MCMV mutant expressing dnCul4A was significantly impaired in its replication. Nevertheless, it is not possible to formally rule out additional effects of MLN4924 beyond CRL regulation.

The antiviral effect of MLN4924 was observed in cells harbouring genetic deletions comprising of type I IFN genes (Vero cells), in IFNAR1-deficient fibroblasts and against ΔM27-MCMV. Beside the exploitation of CRLs for the degradation of STAT2, the cytomegaloviral dependency on CRL activity is more complex and multi-layered. Additional proteins which are required for genome amplification and viral replication must be regulated by CRLs or their function must rely on CRL activity.

Using label-free mass-spectrometry, we determined which CMV-encoded proteins were deregulated upon MLN4924 exposure. To our knowledge, this is the first application of label-free mass-spectrometry to study the host and virus proteome of MCMV-infected cells and the first application of proteome profiling to study the impact of an antiviral drug. Consistent with the antiviral activity of the compound, we found numerous viral proteins (including essential proteins) to be significantly down-regulated by MLN4924. Surprisingly, the abundance of the initial wave of virally expressed proteins (MCMV-encoded pIE1 and pIE3 as well as HCMV-encoded pIE1-pp72) was increased upon MLN4924 treatment. It is noteworthy that HCMV pIE2-pp86 behaved differently: at 72 h post infection, the essential HCMV protein pIE2-pp86^34^ was more than 11-fold down-regulated (ANOVA p < 4.6 * 10^−12^) in MLN4924-conditioned cells. Whether the up-regulation of pIE1-pp72 and/or the down-regulation of pIE2-pp86 are the reason (or at least one reason) for the antiviral activity of MLN4924 remains to be elucidated.

MLN4924 also significantly reduced the replication of HSV-1, HSV-2, AdV5 and to a lesser extent the influenza virus PR8. MLN4924 even elicited antiviral activity against a multidrug-resistant HSV-1 isolate, indicating a mode of action which differs from all drugs currently in clinical use. Retroviridae also express proteins which exploit CRLs (e.g. Vif and Vpx) to induce the degradation of cellular restriction factors (e.g. APOBEC3G and SAMHD1). Consistently, MLN4924 treatment has been shown to reduce the HIV infectivity in cells expressing the respective antiviral factors^35^,^36^,^37^,^38^. Our data add a number of clinically relevant members to the list of MLN4924 susceptible viruses. Based on recent findings which indicate that VACV requires Cullin 3 activity^39^, the resistance of VACV against MLN4924, as observed under our experimental conditions, was surprising. A possible interpretation could be that the presence of Cullin 3 might be essential for VACV, but not its NAE- and Nedd8-dependent activity.

In clear contrast to the previously presented finding that EBV compromises Cullin activity^22^, protein abundance and the overall activity of CRLs were found to be preserved in MCMV-infected cells. An assessment of CRL activity, using p21 as a surrogate marker, indicated unperturbed steady-state levels of p21 upon MCMV infection. HCMV was shown to reduce p21 levels in a pUL29/28- and pUL38-dependent manner^40^. Nevertheless, the antiviral activity of MLN4924 against HCMV, HSV-1 and HSV-2 suggests that members of the α- and β-herpesviruses require NAE and CRL activity for their replication. Thus, the impairment of CRL activity by EBV might represent a specific adaptation.

In addition to the basic research aspects of our newly identified CRL-dependent regulation of cytomegaloviral IE proteins, our data have important clinical implications. Our findings highlight NAE and CRLs as druggable host factors for several clinically relevant viruses and argue for a potential applicability of MLN4924 as a treatment option (e.g. for topical treatment against local HSV infections). Future experiments will address which cellular host restriction factor(s) and/or viral protein(s) are directly subjected to CRL-mediated ubiquitination and proteasomal degradation. An understanding of such mechanisms might yield deeper insights into the regulation of protein turn-over within virus-infected cells and will hopefully pave the way for the design of even more specific antiviral drugs.

## Methods

### Cells, cytokines and compounds

MRC-5 fibroblasts (ATCC CCL-171), NIH3T3 (ATCC CRL-1658), NIH3T3:ISRE-luciferase^21^, primary and crisis immortalized IFNAR1-deficient cells (generated from IFNAR1-deficient MEF^41^), crisis immortalized C57BL/6 cells^42^, Vero (ATCC CCL-81), A549 (ATCC CCL-185), CV-1 (ATCC CCL-70) and MDCK cells were grown in DMEM supplemented with 10% (v/v) FCS, streptomycin, penicillin and 2 mM glutamine.

Mouse IFN-α (#12100-1) was purchased from PBL Biomedical Laboratories, New Jersey, USA. MLN4924 was purchased from Active Biochem (#A-1139).

### Viruses, infection conditions and virus titration

WT-MCMV, ΔM27-MCMV, M27HA-MCMV, Δm157-MCMV:*luciferase*, AD169*varL* and AD169*varS* HCMV, HSV-1 strain F, the multidrug- (Acyclovir-, Cidofovir- and Foscarnet-) resistant HSV-1 isolate and HSV-2 have been described^14^,^17^,^21^,^31^,^43^. The clinical HCMV isolate (called HCMV *KiK3* - based on its origin at the Paediatric Clinic 3 [German: Kinderklinik 3]) was isolated from urine and used after 3 *in vitro* passages. H1N1/Puerto Rico/8/34 (PR8) was kindly provided by Christina Ehrhardt (Inst. of Mol. Virology, Muenster, Germany). Vaccinia virus (VACV) strain Western Reserve (WR) was originally provided by Bernard Moss (National Institutes of Health, Bethesda, MD). Human adenovirus 5 (AdV5) was obtained from the ATCC (VR-5).

For the construction of Δm157-MCMV expressing reporter genes (tdTomato, eGFP, dsRed-Mito) or dn Cul4A, an frt-site-flanked fragment encompassing the HCMV-derived major IE promoter/enhancer (MIEP) in front of the respective gene was introduced into a recombinant MCMV bacterial artificial chromosome (already harbouring an frt-site instead of the m157 CDS) by flp-mediated recombination. The generation of AD169*varL*:*eGFP* HCMV based on the pAD169-BAC2^15^. An frt-site-flanked fragment encompassing the *eGFP* gene controlled by the MCMV MIEP was introduced into AD169*varL*Δgpt (frt site instead of the *gpt* gene of the BAC cassette) by flp-mediated recombination. Recombinant CMVs were reconstituted by transfection (Superfect, Qiagen) of BAC DNA into permissive fibroblasts. CMV infection was enhanced by centrifugation at 800 g for 30 min. Viral titers were determined by standard plaque/foci titration (MCMV and HCMV) or TCID50 assay (12 rows per determination) in case of VSV, HSV-1 and HSV-2.

For the Influenza PR8 experiment, MDCK cells were seeded in 96-well plates and incubated with MLN4924 or DMSO for 30 min before infection with 2000 PFU of PR8. Supernatants were harvested at 4, 24, 48 and 72 h post infection. Viral RNA was isolated using Mini Kit (Qiagen) and viral copy numbers were determined by RT-qPCR as described^44^.

VACV was propagated and titrated in monkey kidney cells CV-1 according to standard methodology. For experimental use, VACV WR virions were purified by two consecutive ultracentrifugation steps through a 36% (w/v) sucrose cushion. CV-1 cells were infected with VACV WR at MOI 0.01 and harvested at indicated time points after infection. For plaque titration assays, serial tenfold dilutions of viral suspensions were prepared and allowed to infect CV-1 cells in DMEM medium containing 2% (v/v) FCS. After 48 h, cells were fixed and stained with 0.1% (w/v) crystal violet solution and plaques were counted to determine plaque forming units (PFU)/ml.

### Immunoblot analysis

Immunoblotting was performed according to standard procedures^41^. Briefly, cells were lysed and equal amounts of protein were subjected to SDS-PAGE and transferred to nitrocellulose membranes. Immunoblot analysis was performed using the following antibodies: mAb anti-ß-actin (Sigma), anti-GAPDH (Santa Cruz sc-25778), rabbit polyclonal anti-HA (Sigma), mAb anti-MCMV-pp89 (CROMA101), mAb anti-p21 (Santa Cruz sc-6246), rabbit polyclonal anti-mSTAT2 (Cell Signaling #4597), rabbit polyclonal anti-hSTAT2 (Santa Cruz sc-476), rabbit polyclonal anti-STAT3 (Santa Cruz sc-482), mAb (3A12) anti-HCMV-pp65 (Abcam), mAb (810R) anti-HCMV-pp72 (Millipore), mAb anti-MCMV-M45 (described in^42^) and MCMV immune serum. Proteins were visualized using peroxidase-coupled secondary antibodies and the ECL chemiluminescence system (GE Healthcare).

### Luciferase assay

Luciferase activity was measured according to the manufacturer’s instructions (pjk) using a microplate luminometer (Mithras LB 943; Berthold).

### Label-free LC-MS/MS analysis

MCMV-infected cells were lysed by sonication (six 10 sec. pulses on ice) in 100 μl lysis buffer (30 mM Tris-HCl, 2 M thiourea, 7 M urea, pH 8.5). After centrifugation (15000 g, 5 min, 4 °C) supernatants were collected and protein concentrations were determined by Bradford assay (Bio-Rad, Hercules, CA). For each sample, 20 μg protein were concentrated by SDS-PAGE (stained with Coomassie Brilliant Blue) and subsequently digested at 37 °C for 16 h using trypsin (SERVA Electrophoresis). For peptide extraction, excised gel bands were covered with 20 μl 50% (v/v) acetonitrile in 0.1% (v/v) trifluoroacetic acid (TFA) and sonicated twice on ice for 10 min. Extracts were dried by vacuum centrifugation and reconstituted in 40 μl 0.1% (v/v) TFA. Peptide concentrations were determined by amino acid analysis on an ACQUITY-UPLC with an AccQ Tag Ultra-UPLC column (Waters) calibrated with Pierce Amino Acid Standard (Thermo Scientific). HCMV-infected cells were lysed by sonication in 50 mM NH_4_HCO_3_ supplemented with 0.1% (w/v) Rapigest (Waters). Samples were centrifuged (14000 g, 10 min, 4 °C), supernatants were collected and protein contents were determined using a Bradford assay. For each sample, 4 μg total protein were tryptically digested in solution. 10 μl 50 mM NH_4_HCO_3_ were added. Disulfide bonds were reduced by adding DTT (final concentration 5 mM) and incubating for 30 min at 60 °C. For the subsequent alkylation, iodoacetamide (final concentration 15 mM) was added and samples were incubated for 30 min in the dark at an ambient temperature. Finally, 2 μl trypsin solution (0.1 μg/μl) were added and the digestion was performed for 16 h at 37 °C. The samples were then acidified with 1.2 μl 10% (v/v) TFA, centrifuged and the supernatant directly used for LC-MS/MS analysis.

For quantitative proteome analysis 350 ng tryptically digested peptides of each sample were analyzed on an Orbitrap Elite or QExactive instrument online coupled to an Ultimate 3000 RSLCnano system (both from Thermo Scientific). Details regarding the chromatographic and mass-spectrometric settings have been published earlier^45^,^46^. For protein and peptide identification, Proteome Discoverer Software (ver. 1.4, Thermo Scientific) was used. Searches were performed with the Sequest HT search algorithm against self-constructed databases. For experiments with MCMV, a database containing UniprotKB-Swissprot entries of *Mus musculus* UniprotKB-Swissprot/TrEMBLE entries of *Murid herpesvirus 1* (72,515 sequences in total, 1,282 virus sequences) was used. For experiments with HCMV, a database containing UniprotKB-Swissprot entries of *Homo sapiens* and HCMV sequences based on recent ribosome profiling data^47^ was used (69,413 sequences in total, 638 viral sequences). Precursor mass tolerances were set at 5 ppm and fragment mass tolerances were 0.4 Da (Orbitrap Elite data) and 0.02 Da (QExactive data). Variable and fixed modifications of amino acids were chosen according to the respective digestion procedure. For in-gel digested samples propionamide (C) and oxidation (M) were chosen as variable modifications. For in-solution digested samples carbamidomethyl (C) was set as a fixed modification, and oxidation (M) as a variable one. Confidence of peptide identification was estimated by a decoy approach implemented in the Percolator function of Proteome Discoverer. Peptide identifications with false discovery rates >1% (q-value N 0.01) were discarded. Protein grouping option was enabled in all analyses. For quantitative data analysis Progenesis LC-MS software was used (ver. 4.0, Nonlinear Dynamics Ltd., Newcastle upon Tyne, UK). A detailed description of each step of quantification has been published earlier^45^,^46^. Briefly, the previously identified peptides were matched to the quantified LC-MS features. For the protein quantification, only peptides unique to one protein within the particular experiment were used. The normalized abundances of proteins (based on the respective abundances of unique peptides) were used to determine fold changes of expression between the experimental groups and statistical evaluation (ANOVA).

### Statistics

Statistical significance comparing multiple mass-spectrometry data sets was tested by applying the ANOVA test. Individual comparisons (e.g. untreated vs. MLN4924-treated) were evaluated with the Student’s t-test: *p < 0.05, **p < 0.01 and ***p < 0.001.

## Additional Information

**How to cite this article**: Le-Trilling, V. T. K. *et al.* Broad and potent antiviral activity of the NAE inhibitor MLN4924. *Sci. Rep.* **6**, 19977; doi: 10.1038/srep19977 (2016).

## Supplementary Material

Supplementary Information

Supplementary Table 1

Supplementary Table 2
