# Supplementary material for: Broad and potent antiviral activity of the NAE inhibitor MLN4924
Source: Sci Rep. 2016 Feb 1;6:19977. doi: 10.1038/srep19977 (PMC4734293; doi:10.1038/srep19977)
Supplement: Supplementary Information [file srep19977-s1.pdf]

Broad and potent antiviral activity of the NAE inhibitor MLN4924

Vu Thuy Khanh Le-Trilling, Dominik A. Megger, Benjamin Katschinski, Christine D. Landsberg, Meike U. Rückborn, Sha Tao, Adalbert Krawczyk, Wibke Bayer, Ingo Drexler, Matthias Tenbusch, Barbara Sitek and Mirko Trilling

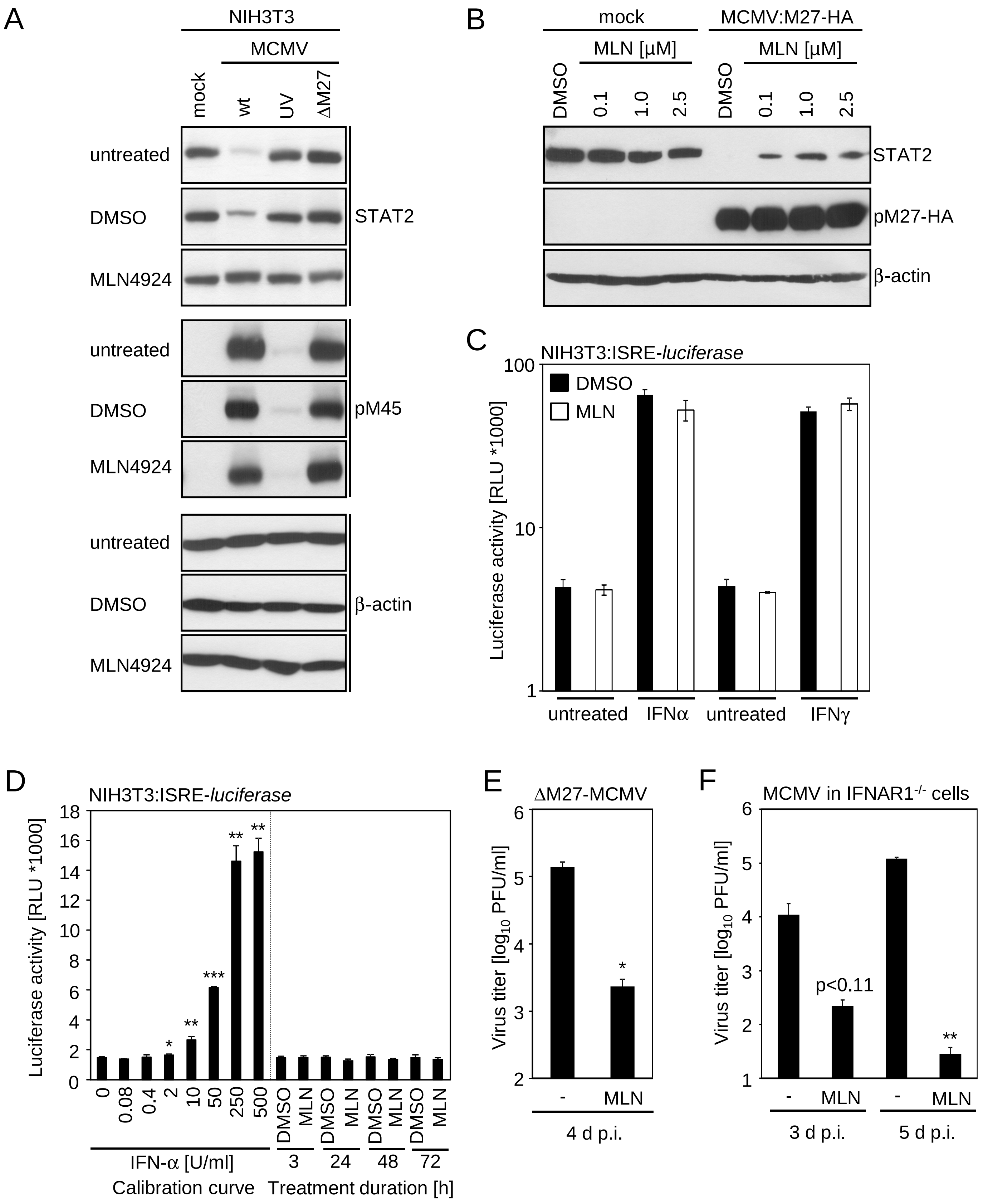

A
NIH3T3
MCMV
mock
wt
UV
ΔM27
untreated
DMSO
MLN4924
STAT2
pM45
β-actin
B
mock
MCMV:M27-HA
MLN [µM]
DMSO
0.1
1.0
2.5
STAT2
pM27-HA
β-actin
C
NIH3T3:ISRE-luciferase
DMSO
MLN
Luciferase activity [RLU *1000]
100
10
1
untreated
IFNα
untreated
IFNγ
D
NIH3T3:ISRE-luciferase
Luciferase activity [RLU *1000]
18
16
14
12
10
8
6
4
2
0
0
0.08
0.4
2
10
50
250
500
DMSO
MLN
IFN-α [U/ml]
3
24
48
72
Calibration curve
Treatment duration [h]
E
ΔM27-MCMV
Virus titer [log10 PFU/ml]
-
MLN
4 d p.i.
F
MCMV in IFNAR1-/- cells
p<0.11
3 d p.i.
5 d p.i.

Supplementary Figure 1: MLN4924 reverts STAT2 degradation in MCMV-infected cells but MLN4924 acts IFN-independently.

(**A**) Untreated, DMSO-treated and MLN4924-treated (2.5 µM) NIH3T3 cells were infected with wt-MCMV, UV-irradiated (9999 J/m^2) MCMV, ΔM27-MCMV or left uninfected. At 24 h post infection, cells were lysed and subjected to immunoblot analysis using indicated antibodies. (**B**) Cells were infected with MCMV:M27-HA (10 PFU/cell) or left uninfected. At 24 h post infection, cells were incubated with DMSO or indicated concentrations of MNL4924. 24 h later, cells were lysed and subjected to immunoblot analysis using indicated antibodies. (**C**) NIH3T3:ISRE-luciferase reporter cells were treated for 5 h with 500 U/ml IFN-α (left panel), IFN-γ (right panel) or left untreated in presence (white bars) or absence (black bars) of 2.5 µM MLN4924. Cells were lysed and luciferase activity was quantified. (**D**) Supernatants of DMSO- or MLN4924-treated (2.5 µM) cells were collected after the indicated time periods of treatment (3, 24, 48 and 72 h post treatment [h p. t.]). Supernatants were applied to IFN-responsive NIH3T3:ISRE-luciferase reporter cells for 5 h. Graded concentrations of murine IFN-α served as a positive control. The IFN calibration curve was measured in duplicates. The supernatants were derived from three independent wells each measured in duplicates (n=3*2). AM with SD is depicted. (**E**) DMSO- or MLN4924-treated (2.5 µM) immortalized C57BL/6 fibroblasts were infected with 0.05 PFU/cell DM27-MCMV. Virus titers were determined 4 d p. i. Three independent replicates were titrated in triplicates (n=3*3). AM with SD is depicted. (**F**) DMSO- or MLN4924-treated (2.5 µM) immortalized IFNAR1-deficient cells were infected with Δm157-MCMV:*eGFP* (0.05 PFU/cell). Virus titers were determined at 3 and 5 days post infection (d p. i.). Three independent replicates were titrated in triplicates (n=3*3). AM with SD is depicted.

A

Protein	Fold Change	Error	ANOVA p value	Unique Peptides
COP9 signalosome complex subunit 1	1.13	±0.28	0.349	3
COP9 signalosome complex subunit 2	0.82	±0.45	0.286	5
COP9 signalosome complex subunit 3	1.21	±1.03	0.369	1
COP9 signalosome complex subunit 4	1.26	±0.47	0.286	3
COP9 signalosome complex subunit 5	1.43	±0.89	0.579	5
COP9 signalosome complex subunit 6	1.11	±0.46	0.904	3
COP9 signalosome complex subunit 8	1.18	±1.23	0.503	3
Cullin-1	1.64	±0.65	0.019	7
Cullin-2	1.28	±0.44	0.170	6
Cullin-3	1.25	±0.27	0.057	16
Cullin-4A	1.43	±0.42	0.035	5
Cullin-4B	1.10	±0.49	0.845	7
Cullin-5	1.32	±0.75	0.853	1
Cullin-7	1.47	±0.90	0.460	2
Cullin-ass. NEDD8-dissociated (Cand) 1	1.39	±0.74	0.282	41
Cullin-ass. NEDD8-dissociated (Cand) 2	1.26	±1.34	0.784	3
DDB1- & CUL4-associated factor (DCAF) 13	0.74	±0.35	0.132	3
DDB1- & CUL4-associated factor (DCAF) 15	0.47	±0.41	0.235	1
DDB1- & CUL4-associated factor (DCAF) 5	1.11	±0.60	0.976	2
DDB1- & CUL4-associated factor (DCAF) 8	1.00	±0.19	0.933	3
NEDD8 ultimate buster (NUB) 1	2.01	±2.69	0.182	1
NEDD8-activating enzyme (NAE) E1	1.12	±0.25	0.386	4
NEDD8-conjugating enzyme Ubc12	0.87	±0.33	0.370	1

B

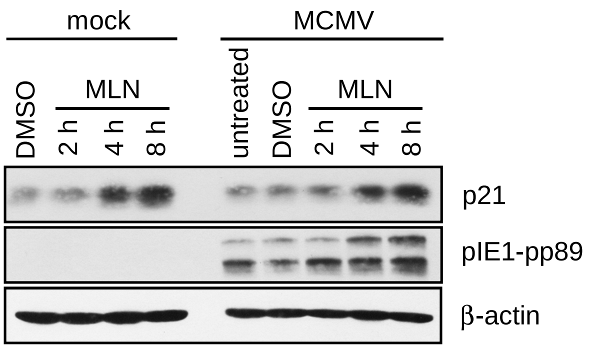

Supplementary Figure 2: Cullin ubiquitin ligases are active within MCMV-infected cells. (**A**) Based on LC-MS data described in Fig. 5A, MCMV-dependent changes in the abundance of indicated proteins - directly or indirectly implicated in CRLs and their activity – were calculated. The fold changes, the deviation (for both conditions [infected and uninfected] the SD were determined individually; based on the higher SD, the error was calculated relative to the indicated fold change), the significance (ANOVA) and the number of quantified peptides are provided. (**B**) Mock- and MCMV-infected (5 PFU/cell; 24 h plus duration of treatment) NIH3T3 cells were treated with DMSO, MLN4924 or left untreated. Cells were lysed after 2, 4 or 8 h of treatment and subjected to immunoblotting using the indicated antibodies.

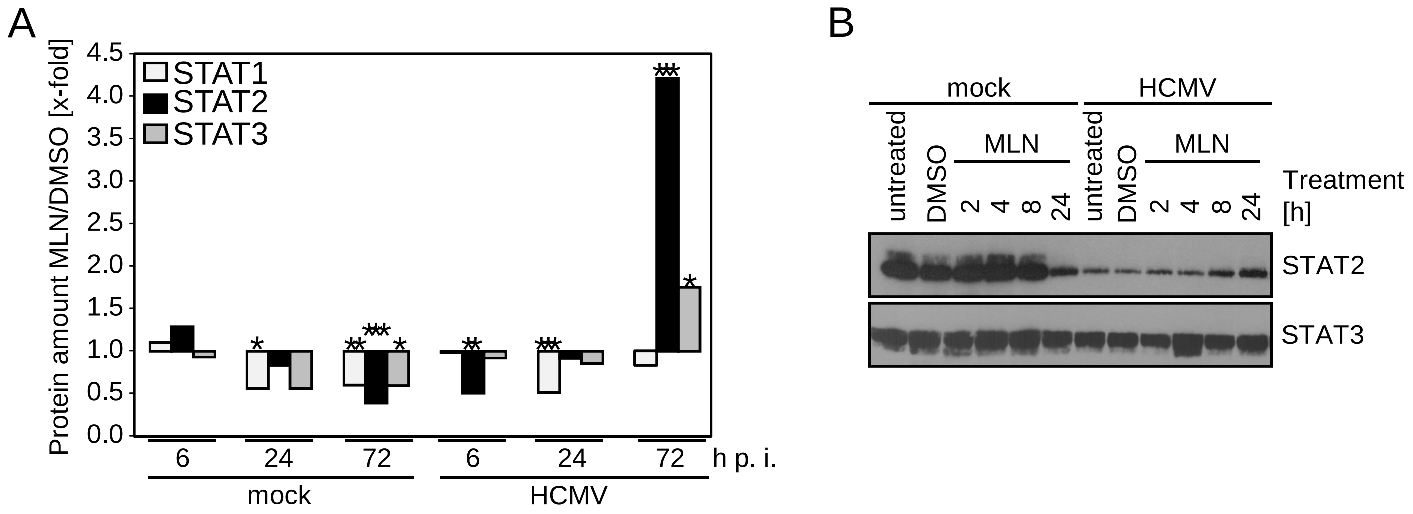

Supplementary Figure 3: MLN4924 reverts the HCMV-induced loss of STAT2
(**A**) Based on the experiment described in Fig. 5B, MLN4924-induced changes in the abundance of STAT1, STAT2 and STAT3 were calculated. (**B**) After 64 h of mock- or HCMV-infection (HCMV AD169*var*L, 3 PFU/cell), cells were treated for indicated time points with DMSO or MLN4924 (1 µM) or left untreated. Cells were lysed and subjected to immunoblot analysis using the indicated antibodies.
